# High-resolution crystal structure of *Streptococcus pyogenes* β-NAD^+^ glycohydrolase in complex with its endogenous inhibitor IFS reveals a highly water-rich interface

**DOI:** 10.1107/S0909049513020803

**Published:** 2013-09-29

**Authors:** Ji Young Yoon, Doo Ri An, Hye-Jin Yoon, Hyoun Sook Kim, Sang Jae Lee, Ha Na Im, Jun Young Jang, Se Won Suh

**Affiliations:** a Department of Chemistry, College of Natural Sciences, Seoul National University, Seoul 151-747, Republic of Korea; b Department of Biophysics and Chemical Biology, Seoul National University, Seoul 151-747, Republic of Korea; c College of Pharmacy, Seoul National University, Seoul 151-742, Republic of Korea

**Keywords:** *Streptococcus pyogenes*, IFS, β-NAD^+^ glycohydrolase, ADP-ribosyltransferase, ARTT loop

## Abstract

One of the virulence factors produced by *Streptococcus pyogenes* is β-NAD^+^ glycohydrolase (SPN). *S. pyogenes* injects SPN into the cytosol of an infected host cell using the cytolysin-mediated translocation pathway. As SPN is toxic to bacterial cells themselves, *S. pyogenes* possesses the *ifs* gene that encodes an endogenous inhibitor for SPN (IFS). IFS is localized intracellularly and forms a complex with SPN. This intracellular complex must be dissociated during export through the cell envelope. To provide a structural basis for understanding the interactions between SPN and IFS, the complex was overexpressed between the mature SPN (residues 38–451) and the full-length IFS (residues 1–161), but it could not be crystallized. Therefore, limited proteolysis was used to isolate a crystallizable SPN_ct_–IFS complex, which consists of the SPN C-terminal domain (SPN_ct_; residues 193–451) and the full-length IFS. Its crystal structure has been determined by single anomalous diffraction and the model refined at 1.70 Å resolution. Interestingly, our high-resolution structure of the complex reveals that the interface between SPN_ct_ and IFS is highly rich in water molecules and many of the interactions are water-mediated. The wet interface may facilitate the dissociation of the complex for translocation across the cell envelope.

## Abstract 2

The crystal structure of the complex between the C-terminal domain of *Streptococcus pyogenes* β-NAD^+^ glycohydrolase and an endogenous inhibitor for SPN was determined at 1.70 Å. It reveals that the interface between the two proteins is highly rich in water molecules.

## 1. Introduction

The gram-positive bacterium *Streptococcus pyogenes* causes a variety of human diseases such as superficial infections (pharyngitis and impetigo) and life-threatening conditions (toxic shock syndrome and necrotizing fasciitis) (Cunningham, 2000 ▶; Sachse *et al.*, 2002 ▶). The virulence of *S. pyogenes* is enhanced by its toxin β-NAD^+^ glycohydrolase (SPN; also known as Nga) (Sumby *et al.*, 2005 ▶). SPN possesses only β-NAD^+^ hydrolytic activity but no ADP-ribosyltransferase or ADP-ribosyl cyclase activity (Ghosh *et al.*, 2010 ▶; Stevens *et al.*, 2000 ▶). SPN is injected across the host cell membrane into the cytoplasm through streptolysin O (SLO), a member of a large family of pore-forming toxins and cholesterol-dependent cytolysins. Once translocated into the cytoplasm of the host cell, SPN contributes to virulence by depleting the intra­cellular NAD pool and producing the potent vasoactive compound nicotinamide, but not by ADP-ribosylation of protein substrates (Ghosh *et al.*, 2010 ▶; Stevens *et al.*, 2000 ▶; Bricker *et al.*, 2005 ▶).

The β-NAD^+^ glycohydrolase hydrolyzes β-NAD^+^, an important cofactor in numerous redox and energy-producing biological reactions, to produce nicotinamide and adenosine diphosphoribose (ADP-ribose) (Tatsuno *et al.*, 2007 ▶; Michos *et al.*, 2006 ▶). Strict β-NAD^+^ glycohydrolases are incapable of further catalysis of the products from the initial reaction (Ghosh *et al.*, 2010 ▶). Other enzymes that can hydrolyze β-NAD^+^ are multifunctional and can be classified into two on the basis of additional reactions (Ghosh *et al.*, 2010 ▶). ADP-ribosyltransferases catalyse the hydrolysis of β-NAD^+^ and the transfer of an ADP-ribose moiety onto target proteins (Holbourn *et al.*, 2006 ▶). ADP-ribosyl cyclases convert β-NAD^+^ into cyclic ADP-ribose, a potent second messenger for calcium mobilization (Karasawa *et al.*, 1995 ▶).

SPN is comprised of two domains. The amino-terminal 190 residues of SPN are required for translocation of SPN into the host cell *via* cytolysin-mediated translocation (CMT) pathway (Ghosh & Caparon, 2006 ▶). The C-terminal domain (residues 191−451) alone is active as the β-NAD^+^ glycohydrolase but it is also indispensible for translocation (Ghosh & Caparon, 2006 ▶; Ghosh *et al.*, 2010 ▶). SPN is also toxic to bacterial cells; therefore *S. pyogenes* encodes the *ifs* gene, which encodes the immunity factor for SPN (IFS) as an endogenous antitoxin (Meehl *et al.*, 2005 ▶; Kimoto *et al.*, 2006 ▶). IFS functions as a competitive inhibitor of the substrate β-NAD^+^ by blocking the active site in the C-terminal domain of SPN. It was reported that increasing concentrations of IFS decreased the rate of β-NAD^+^ hydrolysis, with *K*_I,app_ of 2.0 ± 0.3 n*M* (Meehl *et al.*, 2005 ▶). IFS is essential for the viability of SPN-producing strains of *S. pyogenes* by protecting the bacterium from the toxic β-NAD^+^ glycohydrolase activity of SPN that fail to be secreted (Meehl *et al.*, 2005 ▶).

In order to provide structural details of the interactions of SPN with IFS and thus the inhibition mode, we have employed limited proteolysis to isolate a crystallizable complex of SPN and IFS, which consists of the SPN C-terminal domain (residues 193–451; SPN_ct_) and the full-length IFS (residues 1–161). We have solved the crystal structure of this SPN_ct_–IFS complex by single anomalous diffraction and refined the model at 1.70 Å resolution. The overall complex structure is highly similar to the previously reported structure that was refined at 2.80 Å (PDB entry 3pnt; Smith *et al.*, 2011 ▶). Much higher resolution of the data used in this study allowed us to identify a lot more water molecules bound to the protein complex, in particular at the interface between SPN_ct_ and IFS. Unexpectedly, our higher resolution structure reveals that the interface between SPN_ct_ and IFS is highly rich in water molecules, placing it as one of the wettest protein–protein interfaces. Many interactions between SPN_ct_ and IFS are water-mediated. As an example, the protruding Arg40 of IFS blocks the β-NAD^+^ binding site of SPN_ct_ through extensive water-mediated interactions. If the IFS-inhibited SPN has to dissociate from IFS before the free SPN is translocated across the cell envelope, the wet SPN_ct_–IFS interface may facilitate such dissociation (Ghosh & Caparon, 2006 ▶).

## 2. Materials and methods

### 2.1. Cloning, expression and purification of the SPN_ct_–IFS complex

The contiguous *spn* (SpyM3_0128) gene covering the residues 38–451 and the full-length *ifs* (SpyM3_0129) gene of *S. pyogenes* M3 were PCR-amplified, and cloned into the pET-28b(+) vector (Novagen), using the NdeI/XhoI restriction enzymes. This construct added a hexahistidine-containing 21-residue tag (MGSSHHHHHHSSGLVPRGSHM) at the N-terminus of SPN. The two proteins were co-expressed in *Escherichia coli* Rosetta2 (DE3) cells using Terrific Broth culture medium. Protein expression was induced by 0.5 m*M* isopropyl β-d-thiogalactopyranoside and the cells were incubated for an additional 18 h at 303 K following growth to mid-log phase at 310 K. The cells were lysed by sonication in a lysis buffer [20 m*M* Tris-HCl at pH 8.5, 500 m*M* NaCl, and 5% (*v*/*v*) glycerol] containing 5 m*M* imidazole followed by centrifugation to remove cellular debris. The supernatant was applied to an affinity chromatography column of HiTrap Chelating HP (GE Healthcare). The protein was eluted with the lysis buffer containing 300 m*M* imidazole and the eluted sample was further purified by size-exclusion chromatography using a HiLoad 16/60 Superdex 200 prep-grade column (GE Healthcare). The elution buffer was 20 m*M* Tris-HCl at pH 8.5, 200 m*M* NaCl and 0.1 m*M* tris(2-carboxyethyl)phosphine. We could confirm the complex formation of the two proteins by SDS-PAGE. However, we noticed that the 49 kDa band corresponding to SPN was degraded slowly. Thus, a limited proteolysis experiment was carried out to obtain a proteolysis-resistant core of the complex. After extensive testing of various combinations of proteases (trypsin and chymotrypsin) at different concentrations (at a mole ratio of 1:100, 1:1000 and 1:10000) and incubation time (30 min, 1 h, 3 h, 6 h and 20 h) and temperature (295 K and 310 K), the best condition was established to be α-chymotrypsin (Sigma catalog No. C4129) at a mole ratio of 1:1000 for 20 h at 310 K. After the α-chymotrypsin treatment, the complex was purified by size-exclusion chromatography using a HiLoad 16/60 Superdex 200 prep-grade column.

The selenomethionine (SeMet)-labeled complex protein was expressed and purified as above, except that we used the M9 cell culture medium that contained extra amino acids including SeMet.

### 2.2. Crystallization and X-ray data collection

The protein complex was concentrated to 50 mg ml^−1^ for crystallization using an Amicon Ultra-15 centrifugal filter unit (Millipore). Crystals were grown by sitting-drop vapor-diffusion method at 295 K. Each sitting drop prepared by mixing 1 µl each of the protein solution and the reservoir solution was placed over 100 µl of the reservoir solution. Best crystals of both SeMet-labeled and native SPN_ct_–IFS complex were obtained with the reservoir solution of 20% (*w*/*v*) tacsimate at pH 4.0 and 20% (*w*/*v*) polyethylene glycol 3350. Crystals were transferred to a cryoprotectant solution, which contained 20% (*v*/*v*) glycerol in the reservoir solution. Single-wavelength anomalous diffraction (SAD) data were collected from a crystal of the SeMet-substituted SPN_ct_–IFS complex at 100 K on an ADSC Quantum 315 CCD detector system (Area Detector Systems Corporation, Poway, CA, USA) at the BL-4A experimental station of Pohang Light Source, Korea. Raw data were processed using the program suit *HKL2000* (Otwinowski & Minor, 1997 ▶). The crystal of SeMet-substituted SPN_ct_–IFS complex belongs to the space group *P*1, with unit-cell parameters of *a* = 44.71 Å, *b* = 57.24 Å, *c* = 91.48 Å, α = 72.34°, β = 81.65° and γ = 79.49°. Native X-ray data were collected at 100 K on an ADSC Quantum 270 CCD detector system at the BL-7A of Pohang Light Source. The native crystal belongs to the space group *P*1, with *a* = 43.20 Å, *b* = 56.88 Å, *c* = 89.98 Å, α = 72.96°, β = 90.01° and γ = 82.27°. The presence of two molecules of the complex in the asymmetric unit gives a Matthew’s parameter and solvent fraction of 2.17 Å^3^ Da^−1^ and 43.3%, respectively (Table 1 ▶).

### 2.3. Phasing and refinement

The structure of SPN_ct_–IFS complex was solved by Se SAD phasing. Phase calculation, density modification and initial model building were carried out using *PHENIX AutoSol* and *AutoBuild* (Adams *et al.*, 2010 ▶). *Phenix AutoSol* located all 30 expected selenium atoms of two complex molecules in an asymmetric unit. Subsequent manual model building was conducted using the program *COOT* (Emsley & Cowtan, 2004 ▶) and the model was refined with the programs *REFMAC* (Murshudov *et al.*, 1997 ▶) and *PHENIX* (Adams *et al.*, 2010 ▶), including the bulk solvent correction. 5% of the data were randomly set aside as the test data for the calculation of *R*_free_ (Brünger, 1992 ▶). Water molecules were added using the program *COOT* and were manually inspected. The quality of the refined model was assessed by *MolProbity* (Chen *et al.*, 2010 ▶). Crystallographic and refinement statistics are summarized in Table 1 ▶. The coordinates and structure factors have been deposited in the Protein Data Bank (PDB) under the accession code 4kt6.

## 3. Results and discussion

### 3.1. Preparation of the SPN_ct_–IFS complex and its structure determination

We co-expressed the mature SPN (residues 38–451) and its endogenous inhibitor IFS (residues 1–161) from *S. pyogenes* but we could not crystallize the whole complex, because the SPN component was degraded slowly. Therefore, we optimized the condition of limited proteolysis to isolate a readily crystallizable SPN_ct_–IFS complex. The SPN_ct_–IFS complex consisted of C-terminal residues (193–451) of SPN and all residues (1–161) of IFS. Under our optimized proteolysis condition, the chymotrysin cleavages occurred only before Gly193. The loss of the SPN N-terminal region was supported by mass analysis of trypsin-digested peptide fragments of the denatured complex. Previously, the SPN_ct_–IFS complex was co-expressed with the full-length IFS (residues 1–161) by identifying a C-terminal enzymatically active domain of SPN (residues 191–451) (Smith *et al.*, 2011 ▶). The structure of the SPN_ct_–IFS complex was determined at 2.80 Å (Smith *et al.*, 2011 ▶), with the model accounting for residues 196–445 for both chains A and C of SPN, and residues 1–161 or 2–161 for chains B or D of IFS.

Our crystals of the purified SPN_ct_–IFS complex diffracted to high resolution and allowed us to solve the structure by the Se SAD method. The model of the SPN_ct_–IFS complex was refined to yield *R*_work_ and *R*_free_ values of 19.7% and 23.5%, respectively, for 20.0–1.70 Å data. The model includes 830 residues in two copies of the complex (residues 193–446 of SPN and residues 1–161 of IFS) and 596 water molecules. The C-terminal residues 447−451 of SPN (in both chains A and C) are likely disordered in the crystal. Two independent heterodimeric complexes in the *P*1 unit cell are highly similar to each other, with root-mean-square (r.m.s.) deviations of 0.27 Å for 415 C^α^ atoms (254 residues of SPN_ct_ and 161 residues of IFS) in the model. The two chains of SPN_ct_ in the asymmetric unit are highly similar to each other with an r.m.s. deviation of 0.22 Å for 254 C^α^ atoms; the two chains of IFS in the asymmetric unit are also highly similar to each other with an r.m.s. deviation of 0.22 Å for 161 C^α^ atoms.

Our 1.70 Å structure of the SPN_ct_–IFS complex is highly similar to the previously reported structure determined at 2.80 Å (Smith *et al.*, 2011 ▶), with r.m.s. deviations of 0.62–0.70 Å for 410 C^α^ atoms for each heterodimeric complex. The SPN_ct_ contains the NAD-binding catalytic domain consisting of an α/β fold (Smith *et al.*, 2011 ▶). When we compare the SPN_ct_ part only between the two structures of the SPN_ct_–IFS complex, the r.m.s. deviations are 0.51–0.55 Å for 250 C^α^ atoms. For the IFS part only, the r.m.s. deviations are 0.40–0.46 Å for 160 C^α^ atoms. When we compare the IFS model in our SPN_ct_–IFS complex with the reported structure of the unbound IFS (PDB entry 3qb2; Smith *et al.*, 2011 ▶), the r.m.s. deviations are 7.4–9.3 Å for 156–161 C^α^ atoms. Notable differences between them are found in the N-terminal loop (residues 1–6) with r.m.s. deviations of 20.7–22.1 Å and in the C-terminal region containing the SPN interaction loop 2 (SIL2; residues 139–149) and α7b (residues 150–161) with 15.6–19.7 Å. The N-terminal residues (1–6) of IFS are not involved in the interaction with SPN in the complex but they point in an opposite direction from those of the free IFS.

### 3.2. The interface between SPN_ct_ and IFS is highly rich in water molecules

**Figure 1:**
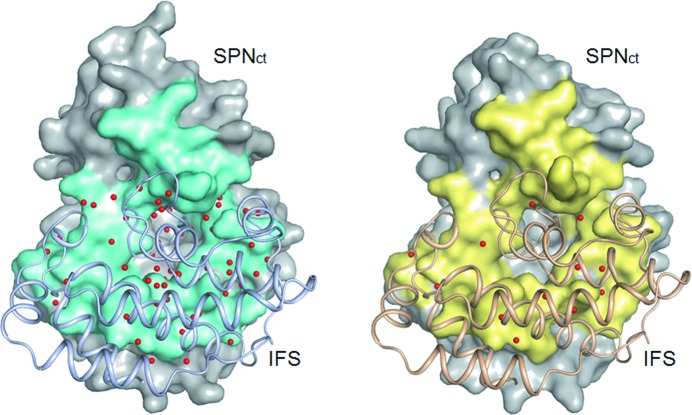
Comparison of the interface waters of two SPN_ct_–IFS complex structures. Left: the 1.70 Å structure reported in this study (PDB entry 4kt6). Right: 2.80 Å structure reported previously by Smith *et al.* (2011 ▶) (PDB entry 3pnt). The A:B interface of the SPN_ct_–IFS complex is shown, representing the SPN subunits as a molecular surface and the IFS subunits as a ribbon. The interfaces are highlighted in cyan and yellow, respectively. Red spheres are the water molecules present at the interface. The interface waters were assigned using the program *AquaProt* (Reichmann *et al.*, 2007 ▶). Structural figures were drawn using *PyMOL* (DeLano, 2002 ▶).

In our high-resolution structure of the SPN_ct_–IFS complex, IFS interacts with SPN_ct_ through numerous hydrogen bonds and electrostatic interactions, many of which are water-mediated. The predominant contacts between SPN_ct_ and IFS involve an α1–α2 loop, α6, α6–β2 loop, α8–β3 loop and α9–β4 loop of SPN_ct_; α1, α2–α3 loop, α5, α7a–α7b loop and α7b of IFS. The complex buries a large surface area at the interface between SPN_ct_ and IFS (3210 Å^2^ and 3280 Å^2^ for A:B and C:D interfaces, respectively). Our higher-resolution (1.70 Å) structure reveals that the interface is very rich in water molecules (Fig. 1 ▶); 67 and 71 water molecules are identified at the A:B and C:D interfaces, respectively. Many of these water molecules are conserved and common to both interfaces. The interface waters have *B*-factors ranging from 20.2 to 50.0 Å^2^ for the A:B interface and from 21.3 to 47.8 Å^2^ for C:D. The mean *B*-factor of interface waters (32.2 Å^2^ for the A:B interface and 33.0 Å^2^ for C:D) is slightly higher than that of non-hydrogen protein atoms (26.9 Å^2^ for A/B chains and 30.0 Å^2^ for C/D chains) but is lower than the overall *B*-factor of other waters (36.0 Å^2^). The previous complex structure was determined at 2.80 Å resolution (Smith *et al.*, 2011 ▶) and it shows essentially identical buried surface areas at the interface (3260 Å^2^ and 3270 Å^2^ for A:B and C:D interfaces, respectively). However, only a small number of water molecules could be located due to insufficient resolution. A total of 153 water molecules were identified per two complex molecules in the asymmetric unit, with only 14 and 10 at the A:B and C:D interfaces, respectively (Fig. 1 ▶). Nearly all of these interface water molecules are present in our higher-resolution structure.

Water is often indispensable for specific recognition of two proteins as an integral part of protein–protein interfaces (Ladbury, 1996 ▶; Levy & Onuchic, 2004 ▶). Analyses of water molecules at the protein–protein interfaces showed that, on average, the interfaces of complexes and homodimers contain about ten water molecules per 1000 Å^2^ of interface area, and crystal packing interfaces, about 15 (Rodier *et al.*, 2005 ▶; Reichmann *et al.*, 2008 ▶). Moreover, interfaces of weak and highly transient complexes contain more waters than found in high-affinity complexes (Rodier *et al.*, 2005 ▶; Reichmann *et al.*, 2008 ▶). In our SPN_ct_–IFS complex structure, ∼21 water molecules are found per 1000 Å^2^, making it one of the wettest protein–protein interfaces. The water-rich interface may be advantageous for facilitating the dissociation of IFS from the complex immediately before translocation across the cell envelope.

**Figure 2:**
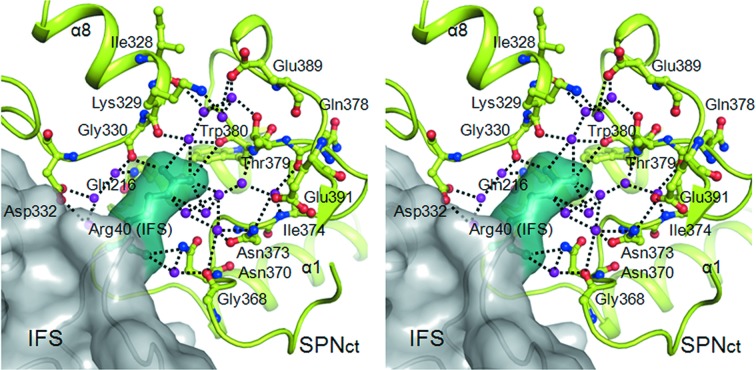
Stereoview of water-mediated interactions between Arg40 of IFS (gray surface) and the active site of SPN (green ribbon). Arg40 of IFS is represented as a ball-and-stick model inside the surface in teal color. Residues of SPN_ct_ interacting with IFS Arg40 are shown as ball and stick. Purple spheres are water molecules and dotted lines denote hydrogen bonds.

Our high-resolution crystal structure of the SPN_ct_–IFS complex reveals that many interactions between SPN_ct_ and IFS are water-mediated. A prominent example is the α2–α3 loop of IFS, which points toward the NAD binding cavity of SPN. Compared with the unbound IFS structure (PDB entry 3qb2; Smith *et al.*, 2011 ▶), the α2–α3 loop of IFS bound to SPN_ct_ is considerably moved toward the active site cavity of SPN in our SPN_ct_–IFS complex, with an r.m.s. deviation of 0.52–0.94 Å for 20 C^α^ atoms. The side-chain of Arg40 on the IFS α2–α3 loop protrudes into the NAD binding cavity of SPN_ct_ (Fig. 2 ▶), blocking the binding of the substrate β-NAD^+^. Arg40 of IFS interacts with SPN_ct_ through extensive water-mediated interactions (Fig. 2 ▶). It makes an extensive water-mediated hydrogen-bond network with the residues located on α2, α8, α8–β3 loop and α9–β4 loop of SPN_ct_ (Gln216 on α2; Ile328 and Lys329 on α8; Gly330 and Asp332 on α8–β3 loop; Gly368, Asn370, Asn373, Ile374, Gln378, Thr379, Trp380, Glu389 and Glu391 on α9–β4 loop).

### 3.3. Structural basis for the lack of ADP-ribosyltransferase activity of SPN

SPN_ct_ was shown to be an atypical member of the ADP-ribosyltransferase superfamily with the characteristic Arg/His (R/H) motif (His273) and the ADP-ribosylating turn–turn (ARTT) motif. The Ser-Thr-Ser (STS) motif of the ADP-ribosyltransferase superfamily is missing in SPN. It has an α-helical linker subdomain, which is absent in other ADP-ribosyltransferase superfamily enzymes (Smith *et al.*, 2011 ▶). The ARTT motif is important for the substrate specificity and recognition of the ADP-ribosyltransferase superfamily (Han & Tainer, 2002 ▶). The Q/E–X–E sequence of the ARTT motif provides the key catalytic glutamic acid to stabilize an oxocarbenium ion intermediate (Han & Tainer, 2002 ▶; Holbourn *et al.*, 2006 ▶). The second Gln or Glu (Q/E), located two positions upstream from the catalytic Glu in the ARTT loop, is essential for the ribosyltransferase activity of ADP-ribosylating toxins. It may be important for recognizing the target residue of substrate proteins (Nagahama *et al.*, 2000 ▶; Wilde *et al.*, 2002 ▶; Han *et al.*, 2001 ▶). It was suggested that the different conformation of the ARTT loop in SPN as well as SPN’s unique α-helical linker sub­domain does not allow accommodation of protein substrates in the canonical mode of other ADP-ribosyltransfereases (Smith *et al.*, 2011 ▶).

**Figure 3:**
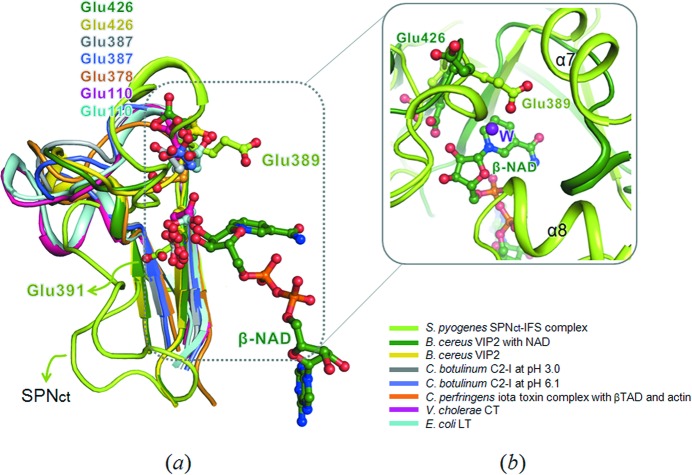
Structural comparison of the second Glu of the ARTT motif between SPN_ct_ and ADP-ribosyltransferases. (*a*) Superimposition of the active site residues (Glu389 and Glu391) of SPN with those of ADP-ribosyltransferases such as *B. cereus* VIP2 complexed with NAD, *B. cereus* VIP2, *C. botulinum* C2 at pH 3.0, *C. botulinum* C2 at pH 6.1, *C. perfringens* iota toxin, *V. cholerae* CT and *E. coli* heat-labile enterotoxin (LT). Other secondary structure elements except for the region containing the ARTT motif were removed for clarity. The regions containing the second Glu of the ARTT motif and β-NAD are marked with a dotted square. (*b*) A slightly different view of the dotted square in (*a*) for the superposition of SPN_ct_ and VIP2 complexed with NAD is shown in the box. A water molecule present in our SPN_ct_ structure is donoted by W (a purple sphere). Secondary structure elements of SPN_ct_ are labeled.

It appears that the distinct side-chain orientation of SPN Glu389 (Fig. 3 ▶), the second Q/E in the ARTT motif, is responsible for the lack of ADP-ribosyltransferase activity in SPN. In our present complex structure, as well as in the previously reported structure (Smith *et al.*, 2011 ▶), the conformation of the ARTT loop of SPN_ct_ is considerably different from ADP-ribosyltransferases. Furthermore, the side-chain of Glu389 is stretched into the interior and is surrounded by α-helices α7 and α8 (Fig. 3*b* ▶). The side-chain orientation of Glu389 is in an almost opposite direction from corresponding residues of ADP-ribosyltransferases (Fig. 3*a* ▶). When we modeled an NAD molecule of *B. cereus* VIP2 (PDB entries 1qs2) into the active site of SPN_ct_ by superimposing the two structures, a water lies in the SPN_ct_ structure between Glu389 and the susceptible glycosidic bond of NAD^+^ (Fig. 3*b* ▶). This water may be activated by Glu389 to act as a nucleophile for the hydrolytic reaction catalyzed by SPN_ct_ (Ghosh *et al.*, 2010 ▶; Robertus *et al.*, 1998 ▶). Unlike Glu389, the side-chain of the catalytic Glu391 of SPN_ct_ overlaps well with the corresponding residues of ADP-ribosyltransferases (Fig. 3*a* ▶).

## Supplementary Material

PDB reference: 4kt6

## Figures and Tables

**Table 1:** Statistics for data collection, phasing and model refinement

Data collection		
Protein name	SeMet-labeled SPN_ct_–IFS complex	SPN_ct_–IFS complex
Data set	SAD (Se peak)	Native
Space group	*P*1	*P*1
Unit-cell lengths (Å)	*a* = 44.71, *b* = 57.24, *c* = 91.48	*a* = 43.20, *b* = 56.88, *c* = 89.98
Unit cell angles (°)	α = 72.34, β = 81.65, γ = 79.49	α = 72.96, β = 90.01, γ = 82.27
X-ray wavelength (Å)	0.9794	1.0395
Resolution range (Å)	50–1.80 (1.83–1.80)†	20–1.70 (1.73–1.70)†
Total/unique reflections	592510/151484‡	308304/82755
Completeness (%)	96.4 (82.2)‡	94.5 (95.1)
Redundancy	3.9 (3.6)‡	3.7 (3.8)
〈*I*〉/〈σ_*I*_〉	36.8 (6.1)‡	33.0 (3.4)
*R*_merge_ (%)§	4.6 (22.1)‡	11.5 (55.4)
Wilson *B*-factor (Å^2^)		34.5

SAD phasing		
Figure of merit (before/after density modification)		0.58/0.75

Model refinement		
Resolution range (Å)		20–1.70
*R*_work_/*R*_free_ ¶ (%)		19.7/23.5
Unique reflections used in *R*_work_/*R*_free_ ¶		74463/4140
No. of non-hydrogen atoms/average *B*-factor (Å^2^)		
Protein		
SPN (residues 193−446), 2 molecules		4064/29.3
IFS (residues 1−161), 2 molecules		2644/27.1
Water		596/35.2
R.m.s. deviations from ideal geometry		
Bond lengths (Å)/bond angles (°)		0.010/1.33
R.m.s. *Z*-scores††		
Bond lengths/bond angles		0.499/0.622
Ramachandran plot (including Gly and Pro)‡‡		
Favored/allowed (%)		98.78/1.22
Rotamer outliers (%)‡‡		0.97

† Values in parentheses refer to the highest-resolution shell.

‡ Values obtained by treating Friedel pairs as separate observations.

§ *R*_merge_ = Σ_*h*_Σ_*i*_|*I*(*h*)_*i*_ − 〈*I*(*h*)〉|/Σ_*h*_Σ_*i*_ *I*(*h*)_*i*_, where *I*(*h*) is the intensity of reflection *h*, Σ_*h*_ is the sum over all reflections, and Σ_*i*_ is the sum over *i* measurements of reflection *h*.

¶ *R* = Σ||*F*_obs_| − |*F*_calc_||/Σ|*F*_obs_|, where *R*_free_ and *R*_work_ are calculated for a randomly chosen 5% of reflections that were not used for refinement and for the remaining reflections, respectively.

†† Values obtained using *Refmac*.

‡‡ Values obtained using *MolProbity*.
